# A Retrospective Study of Adolescent Disc Herniation: Conservative Versus Surgical Management

**DOI:** 10.7759/cureus.82979

**Published:** 2025-04-25

**Authors:** Ashwin Kumar, Manoj K Ramachandraiah, Kavitha R Nagendra, Sagar Venkataraman, Nagakumar J S.

**Affiliations:** 1 Orthopaedics, Sri Devaraj Urs Academy of Higher Education and Research, Kolar, IND; 2 Physiotherapy, Sri Devaraj Urs Academy of Higher Education and Research, Kolar, IND

**Keywords:** adolescent, conservative management, functional outcomes, lumbar disc herniation, oswestry disability scale, surgical management, visual analog scale

## Abstract

Background

Lumbar disc herniation (LDH) is rare in adolescents, with an incidence of 1-5% in individuals under 20 years of age. The condition often manifests as radiculopathy, with or without back pain, posing diagnostic challenges due to differential diagnoses like musculoskeletal disorders. While conservative management is the first-line approach for most patients, surgical intervention is recommended for severe cases or when conservative measures fail.

Objectives

This study aimed to compare functional outcomes in adolescent patients with symptomatic LDH treated conservatively versus surgically.

Methods

A retrospective study was conducted at R.L. Jalappa Hospital, Kolar, India, between May 2023 and April 2024. The study included 44 patients aged 10-19 years with MRI-confirmed LDH. Participants were divided into two groups: conservative management (n = 22) and surgical management (n = 22). Visual analog scale (VAS) and Oswestry Disability Scale (ODS) were used to assess outcomes. Statistical analysis was performed using independent t-tests and chi-square tests, with a significance threshold of p < 0.05.

Results

The mean age was 15.95 ± 0.844 in the conservative group and 16.00 ± 0.816 in the surgical group, with equal gender distribution. VAS scores before and after management showed no significant difference between the groups (p = 0.898 and p = 1.000, respectively). ODS scores after management were significantly better in the surgical group (p = 0.024). Complications were minimal and comparable (p = 0.709).

Conclusion

Both conservative and surgical management effectively reduced pain in adolescent LDH patients, but surgical management demonstrated superior functional outcomes. Complication rates were low and comparable. Further studies with larger sample sizes and longer follow-up are recommended.

## Introduction

Lumbar disc herniation (LDH) in the adolescent population represents a relatively uncommon but clinically significant spinal condition that presents unique diagnostic and therapeutic challenges. While predominantly observed in adults, the incidence of symptomatic disc herniation in individuals aged 10-19 years warrants careful consideration due to its potential impact on growth, development, and long-term functional outcomes [1]. The etiopathogenesis of adolescent LDH differs from adult-onset disease, with developmental, genetic, and biomechanical factors playing more prominent roles than degenerative processes [2]. Unlike adult LDH, adolescent disc herniation often occurs against a background of structurally sound discs with higher water content, potentially influencing both presentation and management strategies [3,4].

The clinical manifestation of LDH in adolescents frequently includes low back pain with radicular symptoms, though atypical presentations may lead to diagnostic delays [5]. Compared to adult counterparts, adolescents with LDH generally demonstrate a more favorable natural history, with some studies reporting higher rates of spontaneous regression and symptomatic improvement [6].

Management strategies for adolescent LDH fall broadly into two categories: conservative and surgical. Conservative approaches typically include physical therapy, activity modification, analgesics, and, in selected cases, epidural steroid injections. This non-operative pathway aims to alleviate symptoms while preserving spinal integrity during a critical developmental period. The efficacy of conservative management in adolescents has been documented in several studies, with resolution rates ranging from 40% to 90% depending on patient selection and outcome measures employed [7].

Surgical intervention becomes necessary when conservative measures fail to provide adequate symptom relief or in cases of progressive neurological deficits [6,8]. Surgical options range from traditional open discectomy to minimally invasive procedures such as microdiscectomy and percutaneous endoscopic discectomy [8,9].

The decision between conservative and surgical management represents a critical juncture in the treatment algorithm for adolescent LDH. Several comparative studies have attempted to evaluate outcomes between these two approaches, with sometimes conflicting results [10,11]. Factors influencing treatment selection include symptom severity, duration, radiographic findings, functional impairment, and patient/family preferences. Standardized assessment tools such as the visual analog scale (VAS) and Oswestry Disability Index (ODI) provide objective measures for evaluating functional outcomes and treatment efficacy [12,13].

Long-term outcomes following adolescent LDH treatment remain an area of ongoing research. Studies by Lagerbäck et al. suggest generally favorable long-term results following surgical intervention, though concerns regarding adjacent segment degeneration and recurrence persist [14]. The impact of early intervention on future spinal health remains incompletely understood, necessitating careful consideration of both immediate symptom relief and potential long-term consequences when formulating treatment plans [15,16]. This study aims to compare the functional outcomes between adolescent patients with symptomatic LDH treated conservatively and surgically, providing evidence to inform clinical decision-making for this unique patient population.

## Materials and methods

Study design

This retrospective study was conducted at R.L. Jalappa Hospital, Kolar, India, between May 2023 and April 2024.

Study population and sample size

Medical records of patients aged 10-19 years with MRI-confirmed LDH were reviewed. Patients aged 10-19 years and MRI evidence of LDH treated conservatively or surgically for at least six months were included in the study. Lumbar disc disease, such as spondylolisthesis, congenital malformation, spinal infection, inflammation, or neoplastic pathology, and previous spinal surgeries or posterior spinal stabilization were excluded from the study. A total of 44 patients were included, with 22 in the conservative treatment group (group A) and 22 in the surgical treatment group (group B). Data retrieved from the patient’s medical records were thoroughly analyzed with at least six months of follow-up.

Study measures

The aim of the study is to compare the functional outcomes between adolescent patients with symptomatic LDH treated conservatively and surgically. The VAS and Oswestry Disability Scale (ODS) were used to evaluate functional outcomes.

Ethics statement

Ethical clearance was received from the Institutional Ethical Committee of Sri Devaraj URS Medical College, Kolar, India (approval number SDUAHER/KLR/R&D/CEC/S/PG/82/2024-25) to conduct the research.

Statistical analysis

Data was entered into MS Excel and analyzed using IBM SPSS Statistics for Windows, version 25.0 (IBM Corp., Armonk, NY). Continuous variables were summarized as mean, standard deviation, median, minimum, and maximum. Categorical variables were presented as frequencies and percentages. The independent t-test was used to test the significance of quantitative variables between two groups. The chi-square test was the test of significance for categorical data. A p-value of <0.05 was considered statistically significant.

## Results

In the present study, in the conservative management group, eight (36.4%) patients were aged 15 years, while in the surgical management group, eight (36.4%) patients were aged 16 years. The mean age was 15.95 ± 0.844 in the conservative management group and 16.00 ± 0.816 in the surgical management group. There was no significant difference in age distribution between the two groups (p = 0.857). Gender distribution was identical in both groups, with 11 (50.0%) males and 11 (50.0%) females, and no significant difference was observed (p = 1.000) (Table 1).

**Table 1:** Profile of subjects in two groups (n = 44) The Pearson chi-square tests and independent samples test were used, and significance was set at a p-value of <0.05.

Variables	Group				Test statistic	p-value
	Conservative management (n = 22)		Surgical management (n = 22)			
	n	%	n	%		
Age						
15	8	36.4%	7	31.8%	χ² = 0.133	0.936
16	7	31.8%	8	36.4%		
17	7	31.8%	7	31.8%		
Mean + SD	15.95	0.844	16	0.816	t = -0.181	0.857
Gender						
Female	11	50.0%	11	50.0%	χ² = 0.000	1.000
Male	11	50.0%	11	50.0%		

In the present study, the mean VAS score before management was 72.95 ± 15.70 in the conservative management group and 73.55 ± 14.52 in the surgical management group. After management, the mean VAS score reduced to 44.64 ± 20.71 in the conservative management group and 44.64 ± 20.12 in the surgical management group. There was no statistically significant difference in VAS scores between the two groups before management (p = 0.898) or after management (p = 1.000) (Table 2).

**Table 2:** VAS score comparison between two groups before and after management (n = 44) The independent samples test was used, and significance was set at a p-value of <0.05. VAS, visual analog scale

Variables	Group						Test statistic	p-value
	Conservative management (n = 22)			Surgical management (n = 22)				
	Mean	SD	Median	Mean	SD	Median		
VAS before	72.95	15.70	71	73.55	14.52	74	t = -0.129	0.898
VAS after	44.64	20.71	37	44.64	20.12	47	t = 0.000	1.000

In the present study, the mean ODS score before management was 54.32 ± 14.89 in the conservative management group and 57.64 ± 13.69 in the surgical management group. After management, the mean ODS score decreased to 41.50 ± 14.25 in the conservative management group and 32.64 ± 10.60 in the surgical management group. There was no statistically significant difference in ODS scores before management (p = 0.446). However, a significant difference was observed after management, favoring the surgical management group (p = 0.024) (Table 3).

**Table 3:** ODS score comparison between two groups before and after management (n = 44) The independent samples test was used, and significance was set at a p-value of <0.05. ODS, Oswestry Disability Scale

Variables	Group						Test statistic	p-value
	Conservative management (n = 22)			Surgical management (n = 22)				
	Mean	SD	Median	Mean	SD	Median		
ODS before	54.32	14.89	55	57.64	13.69	59	t = -0.770	0.446
ODS after	41.50	14.25	39	32.64	10.60	33	t = 2.333	0.024*

In the present study, mild complications were observed in five (22.7%) subjects in the conservative management group and four (18.2%) subjects in the surgical management group. The majority of subjects had no complications, with 17 (77.3%) subjects in the conservative management group and 18 (81.8%) subjects in the surgical management group. There was no statistically significant difference in the incidence of complications between the two groups (p = 0.709) (Table 4).

**Table 4:** Complications comparison between two groups (n = 44) The Pearson chi-square tests were used, and significance was set at a p-value of <0.05.

Variables	Group				Test statistic	p-value
	Conservative management (n = 22)		Surgical management (n = 22)			
	n	%	n	%		
Complications						
Mild	5	22.7%	4	18.2%	χ² = 0.137	0.709
None	17	77.3%	18	81.8%		

## Discussion

This retrospective study compared functional outcomes between adolescent patients with symptomatic LDH treated conservatively and surgically. The analysis revealed comparable VAS score reductions in both treatment modalities, while surgical management demonstrated superior outcomes in ODS scores with minimal complications across both groups. These findings offer valuable insights into the optimal management of adolescent LDH.

The demographic characteristics of our patient cohort align with previous epidemiological studies of adolescent disc herniation. The mean age of 15.95 and 16.00 years in conservative and surgical groups, respectively, corresponds with observations by Karademir et al. (2017), who noted peak incidence in the mid-adolescent years of 15-17 [5]. This age distribution likely reflects the biomechanical stresses that coincide with growth spurts during this developmental stage. Unlike studies reporting male predominance (Elsamea et al., 2018) [4], our study demonstrated an equal gender distribution, possibly attributable to the increasing participation of female adolescents in sports and physical activities in contemporary society.

Our findings of equivalent pre-intervention pain intensity across both treatment groups (VAS scores of 72.95 and 73.55) highlight the substantial symptom burden experienced by adolescents with disc herniation. These baseline values exceed those reported by El-Qadi et al. (2023) in their comparative study (mean VAS 62.4 in conservative and 64.8 in surgical groups, n = 45), suggesting potentially more severe presentations in our samples [7]. The comparable post-intervention VAS scores (44.64 in both groups) indicate that both conservative and surgical approaches effectively alleviated pain, with no statistical superiority demonstrated for either modality. This finding is consistent with Weinstein et al. (2006), who reported similar pain reduction trajectories in both conservative and surgical cohorts in the landmark SPORT trial, albeit in an adult population (n = 501) [11].

The equivalent pain reduction observed in our study challenges the perception that surgical intervention necessarily provides superior analgesia in the adolescent population. This finding supports the concept that natural disc resorption processes may be more robust in adolescents than adults, as proposed by Dang and Liu (2010) in their comprehensive review [6]. They suggested that the higher water content and increased vascularity of adolescent discs might facilitate spontaneous regression of herniated material, potentially explaining the efficacy of conservative management in pain reduction.

However, the significant difference observed in post-treatment ODS scores (41.50 for conservative versus 32.64 for surgical management, p = 0.024) reveals an important distinction in functional outcomes. This disparity suggests that while pain relief may be achieved through either approach, functional restoration appears more complete following surgical intervention. Fairbank and Pynsent (2000) established that a 10-point difference in ODS scores represents a clinically meaningful improvement in function, indicating that the approximately nine-point advantage observed in our surgical cohort likely translates to tangible benefits in daily activities and quality of life [12].

This functional superiority of surgical management aligns with findings from Durham et al. (2000), who reported excellent functional outcomes in 26 (95%) pediatric patients who were treated surgically at long-term follow-up [16]. Similarly, Lagerbäck et al. (2019) demonstrated sustained functional improvements following discectomy in young patients (n = 151) with a mean follow-up of 7.3 years [14]. Our findings contribute to this evidence base by directly comparing functional outcomes between treatment modalities within a contemporaneous cohort.

The mechanism underlying this functional disparity despite equivalent pain reduction remains speculative. One possibility is that surgical decompression more completely relieves nerve root impingement, allowing for more rapid neurological recovery and functional improvement. Wang et al. (2013) proposed that the resiliency of neural structures in adolescents enables substantial recovery following decompression, potentially explaining the superior functional outcomes in surgical patients [8]. Additionally, Raghu et al. (2019) suggested that earlier resolution of mechanical compression through surgery might prevent maladaptive movement patterns that could persist despite pain reduction in conservatively managed patients [15].

Regarding complications, our observation of mild complications in five (22.7%) patients of the conservative group and four (18.2%) patients of the surgical group was found, with no statistically significant difference between groups (p = 0.709), which contradicts some previous literature suggesting higher complication rates with surgical intervention. For instance, Wei et al. (2021) reported complication rates of 4-19% following various surgical procedures for LDH in their network meta-analysis [9]. Our relatively lower surgical complication rate may reflect advancements in surgical techniques and the specialized pediatric surgical expertise available at our institution. This finding is particularly noteworthy given historical concerns about the potential risks of surgical intervention in the developing spine.

The equivalent complication profile between conservative and surgical approaches in our study challenges the conventional risk-averse mindset that often favors conservative management in the pediatric population. Historically, as noted by Ozgen et al. (2007), surgical intervention for adolescent disc herniation was reserved for cases with severe or progressive neurological deficits, based partly on concerns about surgical morbidity [1]. Our findings suggest that modern surgical approaches may carry similar risk profiles to prolonged conservative management, potentially shifting the risk-benefit analysis in treatment selection.

The observed patterns of clinical improvement in our study can be contextualized within the unique pathophysiology of adolescent disc herniation. Unlike adult disc degeneration, which typically involves multiple levels and chronic degenerative changes, adolescent herniation often presents as an acute, single-level pathology in relatively healthy discs (Grobler et al., 1979) [3]. This distinct pathophysiology may explain why both conservative and surgical approaches demonstrated substantial efficacy in our cohort, as the underlying disc health remains relatively preserved compared to adult counterparts.

Our findings also align with emerging evidence suggesting that adolescent disc herniation represents a distinct clinical entity from adult disc disease. Jiang et al. (2023) identified unique MRI characteristics in juvenile disc herniations (n = 84), including more prominent posterior ring apophyseal involvement and less degenerative changes compared to adults [2]. These distinct radiographic features may reflect different underlying pathophysiologies, potentially influencing treatment responses. The comparable pain reduction observed across both treatment groups in our study supports the notion that adolescent discs may possess greater intrinsic healing capacity than previously recognized.

When considering the timing of intervention, our study did not evaluate the influence of symptom duration on outcomes. Nevertheless, several authors have suggested that prolonged conservative management may compromise ultimate functional recovery in adolescents. Cahill et al. (2010) reported better outcomes in pediatric patients (n = 60) undergoing early microdiscectomy compared to those with prolonged preoperative symptoms, highlighting the potential importance of timely intervention [17]. The superior functional outcomes observed in our surgical cohort might partially reflect the benefits of definitive early mechanical decompression, though this hypothesis requires further investigation with prospective designs.

It is worth noting that our findings diverge somewhat from Gugliotta et al. (2016), who reported marginally better outcomes with surgical management at six months but equivalent outcomes at 12 months in their prospective cohort study (n = 370) [10]. This discrepancy may reflect differences in patient populations, with their adult cohort potentially demonstrating different natural history patterns compared to our adolescent sample. Additionally, their cohort included a higher proportion of patients with longer symptom duration, potentially influencing the recovery trajectories observed.

The equivalent pain reduction but superior functional improvement in our surgical cohort raises interesting questions about the relationship between pain and function in adolescent disc herniation. Delgado et al. (2018) demonstrated that pain intensity and functional impairment, while related, represent distinct clinical constructs that may respond differently to therapeutic interventions [13]. Our findings support this conceptual distinction and suggest that a comprehensive assessment of adolescent disc herniation should incorporate both pain and functional measures to capture the full spectrum of clinical outcomes. 

Limitations

Despite these encouraging outcomes, the study had few limitations. The retrospective design and relatively small sample size may limit the generalizability of the findings. Furthermore, just one center was used for the study, which could have introduced selection bias. To evaluate long-term problems and functional outcomes, longer follow-up is required. Future research with larger sample sizes and longer follow-up durations is warranted to better delineate the indications for each treatment modality.

## Conclusions

The findings of the present study indicate that while both conservative and surgical management are effective in reducing pain, surgical management may offer superior functional outcomes. Complications are minimal and comparable between the two approaches. Future research with larger sample sizes and longer follow-up durations is warranted to better delineate the indications for each treatment modality.
